# Trust in haptic assistance: weighting visual and haptic cues based on error history

**DOI:** 10.1007/s00221-017-4986-4

**Published:** 2017-05-22

**Authors:** Tricia L. Gibo, Winfred Mugge, David A. Abbink

**Affiliations:** Department of BioMechanical Engineering, Faculty of 3 mE, Delft University of Technology, Mekelweg 2, 2628 CD Delft, The Netherlands

**Keywords:** Cue weighting, Sensory integration, Haptics, Haptic assistance, Error history

## Abstract

To effectively interpret and interact with the world, humans weight redundant estimates from different sensory cues to form one coherent, integrated estimate. Recent advancements in physical assistance systems, where guiding forces are computed by an intelligent agent, enable the presentation of augmented cues. It is unknown, however, if cue weighting can be extended to augmented cues. Previous research has shown that cue weighting is determined by the reliability (inversely related to uncertainty) of cues within a trial, yet augmented cues may also be affected by errors that vary over trials. In this study, we investigate whether people can learn to appropriately weight a haptic cue from an intelligent assistance system based on its error history. Subjects held a haptic device and reached to a hidden target using a visual (Gaussian distributed dots) and haptic (force channel) cue. The error of the augmented haptic cue varied from trial to trial based on a Gaussian distribution. Subjects learned to estimate the target location by weighting the visual and augmented haptic cues based on their perceptual uncertainty and experienced errors. With both cues available, subjects were able to find the target with an improved or equal performance compared to what was possible with one cue alone. Our results show that the brain can learn to reweight augmented cues from intelligent agents, akin to previous observations of the reweighting of naturally occurring cues. In addition, these results suggest that the weighting of a cue is not only affected by its within-trial reliability but also the history of errors.

## Introduction

Humans are capable of combining multiple redundant sensory cues to interpret and interact with the world. Many perceptual decision studies have shown that cue integration can occur within a sensory modality (e.g. various visual cues or haptic cues) (Landy et al. 1995; Drewing and Ernst 2006; Mugge et al. 2009) and across modalities (e.g. auditory, visual, and haptic cues) (Ernst and Banks 2002; Alais and Burr 2004). Between the visual and haptic sense, multisensory integration has been observed when estimating object size (Ernst and Banks 2002; Gepshtein and Banks 2003; Helbig and Ernst 2008) and shape (Helbig and Ernst 2007). Beyond the perceptual domain, cue integration has been observed in sensorimotor control tasks, with the use of visual and proprioceptive cues to locate hand position (van Beers et al. 1996; 1999), visual, proprioceptive, and vestibular information to control posture (Peterka 2002; Cullen 2012), and Bayesian inference of prior statistical information and sensory feedback to perform a reaching task (Kording and Wolpert 2004). In many instances, humans integrate cues in a near-optimal manner, producing an estimate with maximum likelihood (ML) or minimum variance (Ernst and Bulthoff 2004). In this model, individual estimates from cues are linearly integrated, with the weight of each cue estimate based on its reliability.

A majority of cue weighting studies focus on cue reliability, inversely related to the perceptual uncertainty (noise) of a cue at a given moment, while assuming that each cue is accurate (Rohde et al. 2015). The uncertainty of a cue can be affected by various sources of internal noise, such as sensory noise or neural noise in the human. In the visual system, for example, the inherent reliability of visual cues can be affected by spatial direction and viewing angle (van Beers et al. 1996, 2002; Knill and Saunders 2003; Gepshtein and Banks 2003). Stimulus noise (e.g. visual blur or random dots) can also be artificially introduced to increase the uncertainty of a cue (Ernst and Banks 2002; Alais and Burr 2004; Tassinari et al. 2006). In these studies, cue reliability is intrinsic to the stimulus and weighting of the cues can be determined immediately without learning. Alternatively, other studies have shown that the weighting of cues is not always determined solely by its reliability, but can be adaptively updated over time based on experience and feedback. For example, subjects have been shown to reweight conflicting visual cues (e.g. monocular and binocular cues for slant) after experiencing haptic feedback that was consistent with one cue but not the other (Jacobs and Fine 1999; Ernst et al. 2000; Atkins et al. 2001; van Beers et al. 2011). These findings suggest that the reweighting of cues can also be affected by experience (Sato and Kording 2014), as additional feedback reinforces one of the cues over time.

While many studies have elucidated the mechanisms of cue weighting for stimuli that occur naturally and inherently belong together, it remains unknown whether this behaviour can be extended to *augmented* cues. With advancements in augmented reality for assistance systems, such as superimposed visualizations (Yeh and Wickens 2001) or haptic assistance (Rosenberg 1993; Abbott et al. 2007; Marchal-Crespo and Reinkensmeyer 2008; Abbink et al. 2012; Passenberg et al. 2013), humans must learn to rely on additional cues generated by intelligent agents. Only a few studies have considered cue integration with augmented cues. Note that we distinguish augmented cues from the artificial generation of naturally occurring cues, e.g. force feedback from a haptic device that reproduces the sensation of touching an object (Ernst and Banks 2002). Augmented cues, on the other hand, are less inherent to the interaction, e.g. guidance forces along a path towards an object. Serwe et al. (2011) observed near-optimal cue integration when subjects made reaches to a hidden target based on a noisy visual and augmented haptic (force pulse) cue. However, when subjects used similar cues to determine the direction of the force pulse during movement, this resulted in probabilistic cue switching rather than cue integration (Serwe et al. 2009). Discrepancies over the integration of a cue may be explained by causal inference, as the correspondence between stimuli must be strong enough to provide a reason for cue integration (Kording et al. 2007). For cues that come from a veridical intelligent agent, as in the Serwe et al. studies (2009, 2011), cue weighting is only influenced by the perceptual uncertainty of the cues (e.g. uncertainty in force perception). In addition to perceptual uncertainty from the human, the interpretation of augmented cues may also be affected by errors of the intelligent agent. Here, we focus on the latter, where the intelligent agent generates a cue with *trial*-*by*-*trial random errors*.

In our study, a target-hitting task is used to investigate the weighting of visual and haptic cues, where the haptic cue (providing both perceptual information as well as physical guidance towards the target) originates from an intelligent assistance system with errors. In realistic applications, these errors may be caused by the sensors (e.g. noise, offset, saturation) or models (e.g. mismatch with reality) used by the intelligent system to generate the assistance. Unlike natural haptic cues that people have been exposed to and accumulated knowledge about over their lives, augmented haptic cues will need to be learned in a relatively short period of time. Despite the novelty of the augmented haptic cue, we hypothesize that people will learn to reweight the visual and haptic cues, based on both the relative cue reliability and history of errors over trials.

## Materials and methods

### Subjects

Ten subjects (7 male, age 22–31) participated in the experiment. The experiment was approved by the Delft University of Technology Human Research Ethics Committee and complied with the principles of the Declaration of Helsinki. All subjects gave informed consent prior to participating.

### Experimental setup

**Fig. 1:**
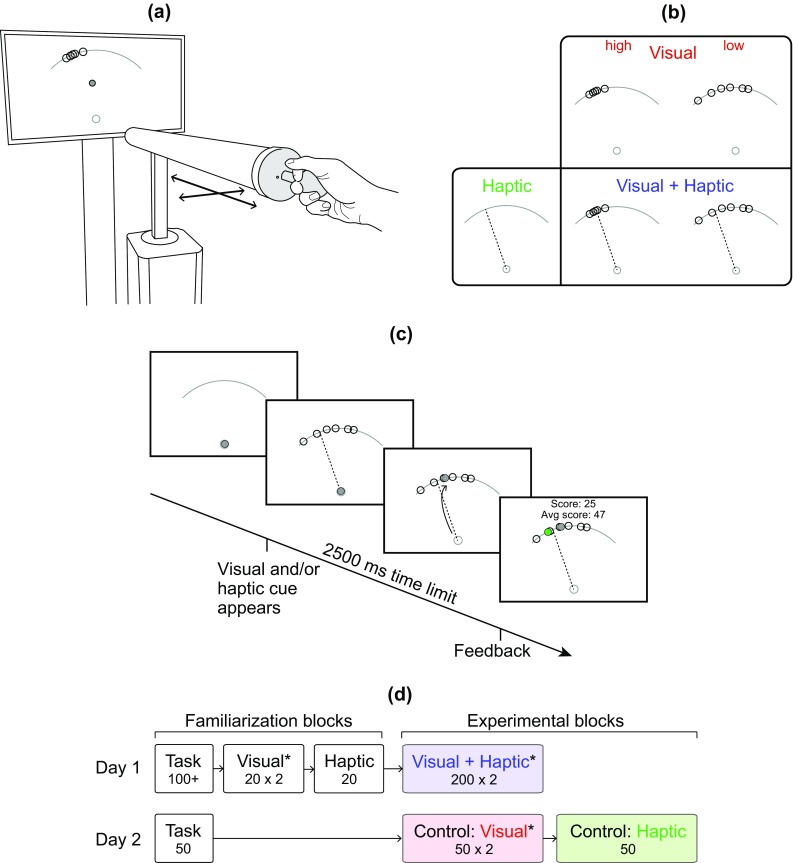
Experimental setup and protocol. **a** Subjects made 2-DOF reaching movements with a haptic device and tried to hit targets, using visual and haptic cues about the target location. **b** The visual cue consisted of six dots around the hidden target (shown upon trial completion when the cursor reached the wall). The haptic cue was a force channel (direction indicated by *dotted line*) that guided movement along a straight path from the start position to a position around the hidden target. There were two levels of visual cue reliability and one level of haptic cue reliability, while the haptic cue also contained trial-by-trial random errors. In the two possible combined cue conditions, subjects’ performance was compared to the performance predicted by the ML estimation model. **c** The order of events in a single trial. **d** On Day 1 of the experiment, subjects’ performance in the combined cue conditions (Visual + Haptic) was measured. From the control experiments for the single cue conditions (Visual, Haptic) on Day 2, their performance in the single cue conditions was calculated. An *asterisk* indicates that the order of the two blocks (high and low visual cue reliability) was counterbalanced across subjects

For the target-hitting task, subjects performed two degree-of-freedom reaching movements while holding onto an admittance-controlled haptic device (HapticMaster, Moog Inc.). Virtual hard constraints confined movements to a horizontal plane. The virtual inertia and damping of the device were set to 2.5 kg and 5 Ns/m, respectively. The device was controlled with a VxWorks RT operating system running at 2048 Hz. All signals, including the handle position (0.001 mm resolution) and force measured at the handle (0.01 N resolution), were recorded by a second controller at 1000 Hz (real-time Bachmann GmbH). The position of the subject’s hand, along with other visual cues during the experiment, was displayed on a monitor (refresh rate 60 Hz, resolution 1920 × 1080 pixels, size 88.5 × 50.0 cm) approximately 140 cm in front of the subject (Fig. 1a). Hand movement in the rightward direction caused the cursor to move right on the screen, and hand movement in the forward direction caused the cursor to move up. The haptic device and one’s arm remained visible.

### Visual and haptic cues

Target locations were chosen randomly and uniformly along the visible curved wall (100° span, 25 cm radius). There were two possible cues providing information about the target location, presented via the visual and haptic sensory modalities. Here, we make the distinction between stimulus noise (perceptual uncertainty within a trial) and stimulus variability (random errors over trials). Manipulations of stimulus noise, as in classical cue weighting studies (Ernst and Banks 2002; Alais and Burr 2004), affect cue reliability. In our study, there were two levels of reliability for the visual cue and one level for the haptic cue. The haptic cue was also affected by stimulus variability, or trial-by-trial random errors with zero-mean. According to classical cue weighting terminology, this type of error does not affect cue reliability because its uncertainty cannot be estimated within one trial (Ernst and Bulthoff 2004; Rohde et al. 2015). Additionally, this type of error does not affect cue accuracy because it is unbiased over trials. To avoid confusion with the classical definitions of cue reliability and accuracy, we will use the term “trial-by-trial random errors”.

The visual cue consisted of six unfilled dots (*N*, 9 mm diameter), chosen randomly and independently from a normal distribution centred on the target location (Fig. 1b). The spread of the distribution controlled the amount of stimulus noise within a trial, resulting in a high (arc length SD *σ*_d_ = 1.2 cm) and low (arc length SD *σ*_d_ = 4.9 cm) level of reliability. The best estimate of the location of the target is the centroid of the dots *x*_Vc_ (Tassinari et al. 2006). If the centroid of the six dots is perfectly determined, the uncertainty in hitting the target is determined by the centroid distribution:

1 $$\sigma_{\text{Vc,t}} = \frac{{\sigma_{\text{d}} }}{\sqrt N }$$

This results in a theoretical target error of 0.5 and 2 cm standard deviation for the high and low reliability cues, respectively.

The haptic cue was a force channel that guided movement along a straight path, producing forces perpendicular to the channel direction. The force increased from 0 to 2 N within 0 to 0.5 cm, then more gradually from 2 to 5 N within 0.5 to 7 cm, and was a constant 5 N outside 7 cm. The force channel could otherwise be described by a piecewise linear stiffness function:

2 $$f = \left\{ {\begin{array}{ll} {k_{1} x, } \hfill &\quad {\left| x \right| < \delta_{1} } \hfill \\ {(k_{1} - k_{2} )\delta_{1} + k_{2} x,} \hfill &\quad {\delta_{1} \le \left| x \right| < \delta_{2} } \hfill \\ {(k_{1} - k_{2} )\delta_{1} + k_{2} \delta_{2} ,} \hfill &\quad {|x| \ge \delta_{2} } \hfill \\ \end{array} } \right.$$

where *k*_1_ and *k*_2_ are 400 and 46 N/m, and *δ*_1_ and *δ*_2_ are 0.5 and 7 cm, respectively. The forces were strong enough to be discernible, yet weak enough to be overridden if desired. The direction of the channel was chosen randomly and independently from a normal distribution centred on the target location (arc length SD *σ*_Hc,t_ = 1 cm), thus determining the error on a given trial. The spread of the distribution controlled the variability of the haptic cue trial-by-trial random error. If the centroid of the force channel *x*_Hc_ is perfectly determined, the theoretical target error has 1 cm standard deviation, thus intermediate between the two visual cues.

### Task

Subjects were instructed to perform quick reaching movements to hit targets. They were told to use the available visual and/or haptic cues to determine the location of the target *x*_t_. Throughout the experiment, subjects stood and grasped the haptic device with their dominant hand. To begin a trial, the subject brought the cursor (5 mm diameter) to the start position (Fig. 1c). Once the cursor was held within the start position for 0.8 s, a visual cue about the target location appeared. The subject then needed to make a reaching movement (25 cm) and try to hit a target, which was located somewhere along the wall. The haptic device simulated a wall (stiffness = 400 N/m, damping = 20 Ns/m), so it was not necessary for subjects to actively bring their hand to rest at the wall. At the end of each trial, the true position of the target (9 mm diameter), the current trial score, and the average score over the current block of trials were displayed. The score was based on the arc length error between the cursor and target, with a maximum score of 100 (0 cm error) that linearly decreased to 0 (2 cm error or greater). When the arc length error was less than 0.6 cm, subjects heard a series of ascending beeps. For all trials, subjects had to complete the task within 2500 ms, starting from when the visual cue appeared; thus, the time limit included both the reaction and movement time. If the trial was not completed within the time limit, or the hand speed dropped below a threshold of 0.015 m/s during movement, a warning message was displayed and a series of descending beeps would sound. The threshold on minimum hand speed was enforced to prevent the motor decision task from becoming more of a perceptual decision task. In preliminary experiments without a minimum hand speed threshold, subjects would sometimes make a ballistic movement in the general direction of the target, stop shortly before the wall, estimate the target location, and then complete their movement. For all other trials, the default sound indicating task completion was one monotone beep.

### Experimental protocol

Due to the trial-by-trial random error of the augmented haptic cue, this experimental protocol differs from that of typical cue integration experiments (Ernst and Banks 2002; Alais and Burr 2004; Rohde et al. 2015), which only focus on cue reliability. The experiment was performed over two consecutive days (Fig. 1d). On Day 1, subjects began with a task familiarization block that consisted of a minimum of 100 trials. Some subjects required more trials before they were able to perform the task sufficiently and consistently. In the task familiarization trials, subjects were shown the true location of the target, to which they needed to reach towards. Next, subjects were informed about the two levels of visual cue reliability. They watched an animation that showed a randomly selected target, followed by six sample dots. Ten examples were shown for each level of visual cue reliability. Subjects then performed visual familiarization trials (20 for each reliability level) to become familiar with performing the task with the uncertain visual cue, in place of the actual target. For all blocks in which the uncertain visual cue was shown, the order of the two visual cue reliability levels was counterbalanced across subjects. To conclude the Day 1 familiarization, subjects were told that they would also receive guidance forces (haptic cue) to help steer them in the direction of the target, but it may not always be correct. Subjects then performed 20 haptic familiarization trials, in which the force channel with trial-by-trial random errors was present and the true location of the target was shown (without visual uncertainty). Here, subjects became familiar with using the forces to help them perform the task, in addition to overriding the forces when they noticed a difference between the direction of the haptic cue and the target location.

Upon completion of the familiarization blocks, subjects performed two combined cue experimental blocks (200 trials each) with both the visual and haptic cues. Within each combined cue experimental block, the visual cue was of either high or low reliability, with the haptic cue present in both. Subjects were instructed to use the visual and haptic cues as they wished, so long as they tried to accurately hit the target and get a high score. After 50 trials, a 30 s break was enforced to prevent boredom and fatigue.

On Day 2, subjects again started with a task familiarization block (50 trials). The following visual control experimental blocks were used to estimate the subjects’ ability to move to the centroid of a set of dots. As previously mentioned, the theoretical standard deviation of hitting the target with the visual cue is given by *σ*_Vc,t_ (Eq. 1), assuming that the centroid of the dots is perfectly determined. However, this is corrupted by additional uncertainty from the human *σ*_h,Vc_, consisting of both noise in estimating the dot centroid location and motor noise. The human uncertainty component was measured by instructing subjects to aim for the centroid of the set of dots. Fifty visual control trials were performed for each level of visual cue reliability, without the haptic cue. Unlike the combined cue experimental blocks of Day 1, subjects did not receive feedback of the true target location or a corresponding score during the control experimental blocks.

Similarly, a haptic control experimental block was performed to estimate the subjects’ ability to follow the haptic cue. The theoretical standard deviation of hitting the target with the haptic cue is given by *σ*_Hc,t_, assuming that the centroid of the force channel is perfectly determined. This, too, is affected by additional uncertainty from the human *σ*_h,Hc_, comprising noise in force perception and motor noise. The human uncertainty component was measured by instructing subjects to follow the centroid of the force channel (50 trials). Since there was no visual cue displayed, the word ‘go’ appeared on the screen to signal when to begin movement.

### Data analysis

The velocity signals were smoothed using a fifth-order, zero phase lag, low-pass Butterworth filter with a cutoff frequency of 10 Hz. All measurements are computed in terms of arc length (along the wall). Trials that were not completed within the time limit, or wherein the hand speed dropped below the set threshold, were omitted from analysis.

**Fig. 2:**
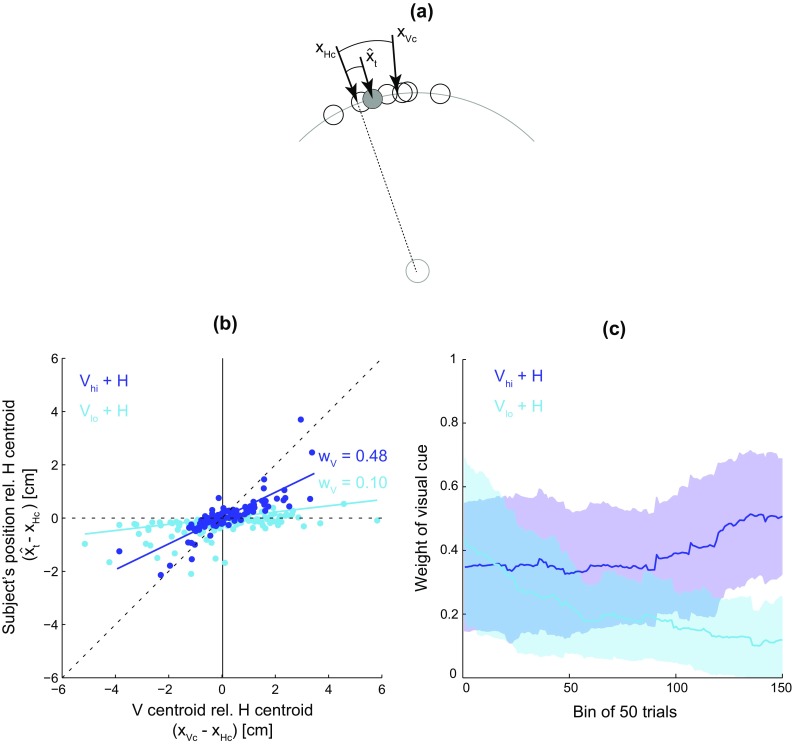
Cue weighting. **a** Weight of the visual cue determined by the relative distance of the subject’s final position $\hat{x}_{\text{t}}$ between the visual centroid *x*_Vc_ (centroid of dots) and haptic centroid *x*_Hc_ (direction of force channel) for each trial. **b** Data from a representative subject, with weight of the visual cue (regressed slope) calculated from the last 100 trials of each combined cue condition. Steeper slope indicates higher reliance on the visual cue. The *dotted diagonal line* indicates reliance on only the visual cue, whereas the *dotted horizontal line* indicates reliance on only the haptic cue. **c** The time course of the visual cue weight averaged over subjects (mean ± standard deviation). The visual cue weight was calculated using a bin of 50 trials over 200 trials of the two combined cue experimental blocks

Data from the last 100 trials of the combined cue experimental blocks on Day 1 were used to calculate the weight that each subject placed on the visual cue relative to the haptic cue. The random generation of the visual and haptic cues results in a discrepancy between the centroid of the dots (best estimate of the target location via the visual cue) and the centroid of the force channel (best estimate via the haptic cue). To determine how strongly the subject relied on the visual cue, the distance between the subject’s final position at the wall and the haptic centroid was plotted versus the distance between the visual and haptic centroids (Fig. 2, similar to Berniker et al. 2010; Sato and Kording 2014). For each subject, a multiple regression model was fit to the data:

3 $$Y = a_{0} + a_{1} X + a_{2} XD$$

$$Y = \hat{x}_{t} - x_{\text{Hc}}$$

$$X = x_{\text{Vc}} - x_{\text{Hc}}$$

$$D = \left\{ {\begin{array}{*{20}c} {0, \quad {\text{high visual cue reliability}}} \\ {1,\quad {\text{low visual cue reliability}} } \\ \end{array} } \right.$$

where $\hat{x}_{\text{t}}$ is the subject’s final position, *x*_Hc_ is the haptic centroid, *x*_Vc_ is the visual centroid, and *D* is a categorical variable depending on the level of visual cue reliability. The visual weight *w*_V_ for the high and low visual cue reliability conditions, as determined by the calculated regressed slopes, is given by *a*_1_ and *a*_1_ + *a*_2_, respectively. A slope close to 1 indicates that the subject relied heavily on the visual cue, whereas a slope near 0 signifies a greater reliance on the haptic cue. A significant interaction term *a*_2_ indicates that the slope is significantly different between the two combined cue conditions. Note that this analysis assumes that the position reached at the end of movement reflects the subject’s belief about where the target is located.

**Fig. 3:**
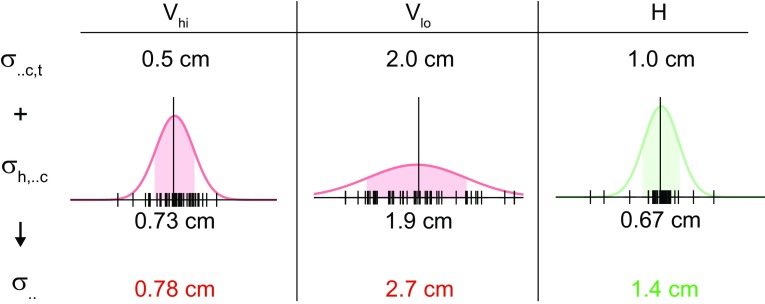
Trial-by-trial target error variability with single cue. The standard deviation of the target error over trials using the visual (high, low reliability) and haptic cue alone $\sigma_{\cdot \cdot}$ was calculated from Eq. 4, which sums the variability resulting from human uncertainty measured in the control experiment $\sigma_{{{\text{h}}, \cdot \cdot {\text{c}}}}$, where $\cdot \cdot$ is either *V* or *H*, and the defined distribution is used to generate the cue $\sigma_{{ \cdot \cdot {\text{c}},{\text{t}}}}$. The distribution used to generate the visual cue affects its reliability within a trial, while the distribution used to generate the haptic cue affects the random errors across trials. Data from the control experiments of a representative subject (*black vertical lines*) show the human uncertainty in locating the visual or haptic centroid (set at zero). The corresponding probability density function of a normal distribution is drawn, with the *shaded* region indicating ± 1 standard deviation

The target error was defined as the subject’s final position relative to the target position, with its standard deviation *σ*_V+H_ calculated over the last 100 trials of each of the two combined cue experimental blocks. For the single cue analysis, the target error standard deviation was calculated from the human uncertainty measured during the Day 2 control experiments ($\sigma_{\text{h,Vc}} , \sigma_{\text{h,Hc}}$) and the defined distributions used to generate the cues ($\sigma_{\text{Vc,t}} , \sigma_{\text{Hc,t}}$). Note that the distribution used to generate the visual cue determines its reliability (spread of dots on a given trial), and the distribution used to generate the haptic cue determines its variable error across trials, but both contribute to the target error variability. Assuming that these factors are independent, the trial-by-trial variability in locating the target with a single cue is calculated by

4 $$\sigma_{..} = \sqrt {\sigma_{{{\text{h}}, \cdot \cdot {\text{c}}}}^{2} + \sigma_{{ \cdot \cdot {\text{c,t}}}}^{2} }$$

where $\cdot \cdot$ is either *V* or *H* (Fig. 3). For each of the two visual control experimental blocks, *σ*_h,Vc_ was determined by calculating the standard deviation of the error of the subject’s final position from the centroid of the dots. Likewise, in the haptic control experiment block, *σ*_h,Hc_ was determined by calculating the standard deviation of the error of the subject’s final position from the centroid of the force channel.

### Maximum likelihood estimation model

The subjects’ performance over trials in the combined cue conditions can be compared to the optimal performance as determined by a maximum likelihood (ML) estimation model. In cue integration studies, ML predictions are typically based on the uncertainty of an estimate (variance of its likelihood function) within a given trial (Rohde et al. 2015). Here, we use the variability in the target location estimate across trials, thus considering both cue reliability and trial-by-trial random errors. Thus, the ML model maximizes the percentage of correct target location estimates, or in other words, minimizes the target error variability over trials.

On a given trial, the ML estimate of target location $\hat{x}_{\text{t}}^{*}$ is the weighted sum of the estimates from the individual cues:

5 $$\hat{x}^{*}_{t} = w_{\text{V}} x_{\text{Vc}} + w_{\text{H}} x_{\text{Hc}}$$

with the weight inversely proportionally to the target error variance with the respective individual cue estimate:

6 $$w_{\text{V}}^{*} = \frac{{1/\sigma_{\text{V}}^{2} }}{{1/\sigma_{\text{V}}^{2} + 1/\sigma_{\text{H}}^{2} }}$$

7 $$w_{\text{H}}^{*} = \frac{{1/\sigma_{\text{H}}^{2} }}{{1/\sigma_{\text{V}}^{2} + 1/\sigma_{\text{H}}^{2} }}$$

The ML estimate also minimizes the target error variability over trials:

8 $$\sigma^{*}_{\text{V + H}} = \sqrt {\frac{1}{{1/\sigma^{2}_{\text{V}} + 1/\sigma^{2}_{\text{H}} }}}$$

Thus, performance in the combined cue conditions can be predicted by the performance achievable with the single cues.

## Results

### Behavioural results: cue weighting

The subjects’ target-hitting performance with different available cues was determined from the combined and single cue experimental trials. Of a total of 3500 trials, 53 trials were omitted from analysis because they were either not completed within the time limit or the hand speed dropped below the set threshold. Of the analysed trials, the average reaction and movement time (mean ± standard deviation) were 1353 ± 130 and 425 ± 105 ms, respectively.

**Fig. 4:**
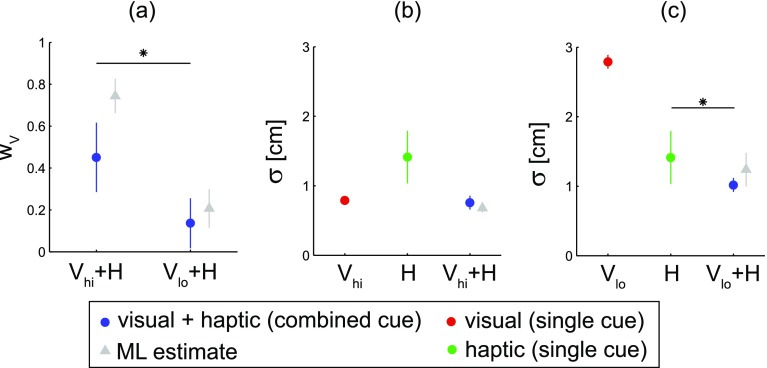
Group data. **a** Weight of visual cue in the two combined cue conditions (mean ± standard deviation). **b**, **c** Trial-by-trial target error variability (standard deviation) for each combined cue condition (*blue*) and the two corresponding single cue conditions (*red* visual, *green* haptic). *Asterisks* represent *p* values <0.05 from comparisons between the combined cue condition and the more reliable of the two single cue conditions. *Grey triangles* show weights and target error variabilities predicted for the combined cue conditions from the single cue data using the ML estimation model for each subject (mean ± standard deviation)

From the two combined cue experimental blocks, where both the visual and haptic cue were present, the weighting of the visual cue was determined. Figure 2b shows the different visual weights for a representative subject, as determined by the regressed slopes (Eq. 3). This trend was observed across subjects (Fig. 4a), with nine of the ten subjects showing a significant difference in the visual cue weight of the two combined cue conditions (Table 1). The weight of the visual cue was greater during the high visual reliability condition compared to the low visual reliability condition (*t*_9_ = 6.7, *p* < 0.001). For these calculations, only the last 100 trials of the combined cue experimental blocks were used, thus allowing sufficient time for the subjects to calibrate their cue weights (Fig. 2c).

**Table 1:** Visual cue weighting for individual subjects

Subject	Visual cue weight *w*_V_		*R*	*a*_2_ *p* value
	*V*_hi_ + *H*	*V*_lo_ + *H*		
1	0.79	0.28	0.85	1.0 × 10^−21^
2	0.55	0.40	0.67	0.084
3	0.37	0.14	0.56	6.8 × 10^−5^
4	0.27	0.034	0.59	2.5 × 10^−12^
5	0.25	0.083	0.37	0.006
6	0.35	0.06	0.55	3.2 × 10^−16^
7	0.41	0.14	0.48	1.4 × 10^−4^
8	0.48	0.10	0.69	3.5 × 10^−15^
9	0.60	0.01	0.77	1.2 × 10^−32^
10	0.62	0.11	0.74	5.0 × 10^−18^

Visual cue weight (slope), correlation coefficient *R*, and *p* value of interaction term *a*_2_ from the multiple regression model (Eq. 3) fit to each subject’s combined cue data

### Behavioural results: target error variability

In addition to cue weighting, the trial-by-trial variability in locating the target was compared between the combined cue and single cue conditions. The variability with either the visual or haptic cue was calculated from the defined distributions used to generate the cues (affecting visual cue reliability and haptic cue random error) and the human uncertainty measured during the control experiments (Fig. 3). For the latter, the measured standard deviation in locating the visual or haptic centroid across subjects was 0.61 ± 0.06 cm (high visual reliability), 1.9 ± 0.1 cm (low visual reliability), and 0.9 ± 0.6 cm (haptic). The variability in the combined cue conditions was directly measured, and then compared with the more reliable of the two single cues. The target error variability with both the high reliability visual cue and haptic cue present was not significantly different than that of the high reliability visual cue alone (*t*_9_ = 1.0, *p* = 0.33) (Fig. 4b). On the other hand, the combination of the low reliability visual cue and haptic cue enabled subjects to locate the target with reduced trial-by-trial variability compared to the haptic cue alone (*t*_9_ = 3.1, *p* = 0.012) (Fig. 4c).

### ML model comparison

Using the maximum likelihood (ML) estimation model, cue weights were predicted for the combined cue conditions, based on the trial-by-trial variability in target error for the single cue conditions of each subject. The decreased visual cue weighting for the combined cue condition with low visual reliability is predicted by the ML model. However, subjects appeared to rely more on the haptic cue compared to the model prediction (Fig. 4a). This over-reliance on the haptic cue was significant for the high visual reliability condition (*t*_9_ = 4.8, *p* < 0.001), but not the low visual reliability condition (*t*_9_ = 1.5, *p* = 0.17). Using the ML cue weights, the trial-by-trial target error variability of the resulting cue combination is only slightly less than that of the more reliable single cue. For the combination of the high reliability visual cue and haptic cue, the predicted decrease in standard deviation compared to the high reliability visual cue was approximately 1.1 mm (Fig. 4b). Subjects were slightly more variable than the ML estimate (*t*_9_ = 9.1, *p* < 0.001). Similarly, the predicted decrease in standard deviation for the combined low reliability visual cue and haptic cue, relative to the haptic cue, was approximately 1.7 mm (Fig. 4c). Interestingly, the subjects’ measured variability in this combined cue condition was lower than the ML estimate (*t*_9_ = 3.9, *p* = 0.004).

Note that the ML estimates described above were calculated based on target error variability, which includes both cue estimate reliability and trial-by-trial random errors (see “Materials and methods” section). Alternatively, the ML estimates could be calculated using only cue estimate reliability (within-trial uncertainty in locating the visual $\sigma_{\text{h,Vc}}$ or haptic $\sigma_{\text{h,Hc}}$ centroid), similar to classical cue weighting studies. For the combined cue conditions, this reliability-based ML estimate results in visual weights of 0.59 ± 0.30 and 0.20 ± 0.17 for high and low visual reliability, respectively. These mean values are closer to the measured values (blue circles in Fig. 4a) than the original ML estimates (Fig. 4a), yet there is much more variability in the reliability-based ML estimates. A regression analysis between the measured and estimated weights reveals a higher correlation when the ML model takes both cue estimate reliability and trial-by-trial random errors into consideration (*R* = 0.71), rather than just reliability (*R* = 0.23). This provides evidence that both within-trial reliability and trial-by-trial variability were used to weight cue estimates.

## Discussion

The results of our study show that subjects learned to estimate the target location by weighting the visual and haptic cue estimates. The haptic information also constituted physical assistance towards the target, generated from an intelligent source, i.e. an assistive system, that contained trial-by-trial random errors centred around the target location. In general, subjects demonstrated cue reweighting and improved or equal performance with both cues compared to single cue performance, similar to what has been previously observed with the integration of naturally occurring cues.

### Cue weighting to improve task performance

#### Similar behaviour for augmented and naturally occurring cues

Our results provide evidence that the integration of sensory information is adaptive and the human brain can learn to incorporate augmented cues. It is conceivable that people may down-weight a cue that is artificially simulated (Campos and Bulthoff 2012), due to a lack of trust or understanding about how the cue was determined. Nevertheless, our subjects learned to trust that the augmented haptic cue, like the visual cue, corresponded to the target position. This assumed causal relationship may have resulted from conscious knowledge due to our instructions about the cues prior to the experiment, and was strengthened as subjects observed sufficient correspondence between the cues during the experiment. Our results are in accordance with those of Serwe et al. (2011), where subjects integrated noisy visual information and an augmented force pulse cue to infer location of a hidden target during reaching movements. In their study, the veridical haptic cue was weighted based on the reliability of the haptic sensory system, whereas in our study, the weighting of the haptic cue was further affected by trial-by-trial random errors in the haptic cue. The adaptive mechanisms of sensory integration are further supported by recent work where monkeys learned to make reaches by optimally combining vision with an artificial proprioceptive cue, delivered via an intracortical microstimulation signal to the brain (Dadarlat et al. 2015). Additionally, Ernst (2007) showed that people are able to learn artificial correlations between naturally occurring cues (object stiffness and luminance) in a perceptual discrimination task. While our experiment did not focus on the time course of cue weights, other experiments that were specifically designed to study the trial-by-trial reweighting of a cue or prior information have also showed that this is a relatively fast learning process, occurring within one experimental session (Berniker et al. 2010; van Beers et al. 2011; Sato and Kording 2014).

#### Effect of cue trial-by-trial random errors on weighting

Furthermore, our results suggest that cue weights can be affected by the error history of a cue, and not just the reliability of its estimate, in order to reduce the error of the combined cue estimate. While the majority of previous work has focused on cue reliability, relatively few studies have directly studied trial-by-trial random errors of a cue. Block and Bastian (2010) had subjects reach to visual targets and proprioceptive targets (designated by the other unseen hand), while altering the bias and variance of the error feedback displayed over trials. Unlike our experiment, their manipulations of the reaching error history had little effect on cue weighting. This discrepancy may be due to the relatively small amount of time that their subjects were exposed to the manipulated error histories (30 trials). Additionally, it may be easier to learn the statistics of the error history of an artificially generated cue, compared to altering that of a familiar, naturally occurring cue (e.g. proprioception).

In other studies, however, the experience-dependent reweighting of cues has been observed, where subjects were presented with inconsistent cues and the accuracy of one was reinforced over time via additional feedback (Jacobs and Fine 1999; Ernst et al. 2000; Atkins et al. 2001; van Beers et al. 2011). It is possible that the brain is capable of learning the uncertainty of a cue’s accuracy and down-weighting the less stable cue (Ernst and Bulthoff 2004). While in these former studies, the feedback always reinforced one of the two conflicting cues, our results suggest that the brain is capable of estimating more complex statistical properties of error history, as feedback of the true target position in our study did not consistently reinforce either the visual or haptic cue. Additionally, cue conflicts in these previous studies were relatively small and subjects remained unaware of any cue discrepancies. Alternatively, our study used larger cue conflicts and unambiguous visual feedback of the true target location. It is possible that conscious effort or increased attention to the cue that better estimated the hidden target location resulted in the observed reweighting. Previous findings have shown that awareness of a cue conflict (Lambrey and Berthoz 2003; Berger and Bulthoff 2009) and explicit instructions to rely on a cue (Block and Bastian 2010) can affect cue weighting, while the consciously ignored cue can still have an effect on the response.

Despite awareness of the cue conflict in our study, which could affect belief about cue correspondence (Kording et al. 2007), subjects still integrated the visual and haptic cues to estimate the target location, rather than basing their decision on a single cue alone. The latter occurs when a cue switching strategy is adopted (Landy and Kojima 2001; Rosas et al. 2005; Serwe et al. 2009). In a cue switching model, the estimate is only based on one cue at a time, where the probability of selecting a cue estimate is proportional to its reliability. After averaging over trials, this can resemble a cue weighting strategy. However, cue switching cannot improve performance relative to single cue performance and would have resulted in an increased trial-by-trial target error variability, which was not observed in our study.

The observed adaptation of cue weighting based on the history of cue error is in accordance with other learning mechanisms that have shown to be sensitive to the statistical properties of errors. In a reaching task where the reliability of the visual feedback of endpoint position was manipulated, subjects were able to learn the variability of the externally imposed noise in <120 trials and appropriately change their pointing strategy (Trommershauser et al. 2005). The rate of error-dependent learning has also been shown to be affected by the statistics of externally imposed visuomotor (Burge et al. 2008) or force perturbations (Smith and Shadmehr 2004). Thus, it appears that the brain is capable of estimating the trial-by-trial variability of extrinsic sources from observations over time in order to improve performance.

#### Maximum likelihood model comparison

The ML model predicted the trends observed in the experimental results for cue reweighting and target error variability, although some discrepancies remain. Subjects showed an over-reliance on the haptic cue, particularly during the combined cue condition with high visual reliability. This discrepancy may result from incorrect estimation of the achievable target-hitting performance with a single cue. In our analysis, the trial-by-trial target error variability with a single cue was used to predict the combined cue performance. Alternatively, we can use the trial-by-trial target error variability with the combined cues to estimate the single cue performance. As such, the free parameters (*σ*_Vhi_, *σ*_Vlo_, *σ*_H_) of the set of non-linear equations (Eqs. 6 and 8) were fit using the function ‘fsolve’ in Matlab (The MathWorks, Inc.). The fitted values for trial-by-trial target error variability *σ*_Vhi_, *σ*_Vlo_, and *σ*_H_ were 1.2, 3.8, and 1.1, respectively. In comparison with the computed values from the control experiments (Fig. 4b, c), the fitted values for both visual cue levels are higher than the corresponding experimental values (0.79 and 2.8 cm), whereas the fitted value for the haptic cue is lower than the experimental value (1.4 cm). This may have resulted from subjects’ underestimation of the reliability of the visual cue. Subjects may have also underestimated the variability of the random errors of the haptic cue, as the appropriate integration of error history is thought to be a slow and difficult (albeit possible) process (Burge et al. 2008; Block and Bastian 2010). Alternatively, the clearly discernible uncertainty of the visual cue (dots in six different directions) compared to the seemingly reliable haptic cue (force channel in one direction) may have caused subjects to over-rely on the latter.

Another reason for this difference may arise from the non-independence of cues. In the combined cue conditions, the visual cue may improve perception of the force channel by providing information about its general direction, thereby increasing the reliability of the haptic cue compared to what was measured in the haptic control block. The predictions of the ML model, however, assume that sensory cues are independent (Ernst and Bulthoff 2004), i.e. the reliability measured in the single cue condition is representative of its reliability in the combined cue condition. Thus, subjects’ misestimation of cue reliability and error history, and the dependence of the haptic cue on visual information, can explain the increased weighting of the haptic cue.

Lastly, predictions from the ML model could be affected if subjects used distinct control strategies in the single and combined cue conditions. In the haptic control block, with only the haptic cue present, subjects likely relaxed their arm to follow the force channel. This contrasts with the combined cue blocks, where subjects sometimes pushed against the force channel. This may have caused the target-hitting performance with the haptic cue to differ between the single cue and combined cue conditions, thus challenging the ML estimates. Nevertheless, performance in the control blocks indicates how well subjects could perform the target-hitting task with a single cue available, irrespective of the control strategy used.

### Experimental protocol limitations

While subjects exhibited cue reweighting in the two combined cue conditions, this did not result in a significant improvement in target-hitting performance relative to single cue performance. The limited reduction in target error variability is predicted by the ML model and can be explained by the design of the experimental cues. When selecting the distributions used to generate the cues, a trade-off exists between amplifying the effect of cue reweighting versus reduction in target error variability. The decrease in target error variability would be the greatest when the target-hitting performance of the two single cues was equal. On the other hand, a larger difference in the target-hitting performance with the single cues makes it easier to detect a change in cue weighting. In our study, we selected the defined distributions from which the cues were generated so as to increase the difference in cue weighting between the two combined cue conditions ($\sigma_{\text{Vhi}} \approx \frac{1}{2}\sigma_{\text{H}} , \sigma_{\text{Vlo}} \approx 2\sigma_{\text{H}}$), thus reducing potential improvements in target error variability.

The design of the single cues also has an effect on the range of discrepancies between the visual and haptic centroids. This range was relatively small (Fig. 2b), particularly for the combined cue condition with the high reliability visual cue. If the discrepancies between the visual and haptic cues are predominantly very small, calculation of the weighting via the regression model becomes difficult and unreliable. While this issue can be addressed by increasing the discrepancies between the cues, an undesirably large spatial discrepancy can cause one cue to be vetoed or completely disregarded (Bulthoff and Mallot 1988; Gepshtein et al. 2005). Thus, the cue discrepancies were kept relatively small to prevent any doubt that the two cues belonged together, particularly for the augmented haptic cue from the intelligent system. Trust in intelligent systems, such as automation, can be influenced by three factors—performance (what it does, e.g. reliability and accuracy), process (how it operates), and purpose (why it was developed) (Lee and See 2004). In our experiment, we wanted to focus on the first aspect, whereas a highly erroneous haptic cue may have caused subjects to doubt the appropriateness or intentions of the assistance (second and third aspects). Future work will investigate the effect of changes in the performance of the haptic assistance, i.e. fluctuating variability of the trial-by-trial random errors, on cue weighting.

Another interesting point of discussion involves the dynamics of the presentation of the cues, although we do not think this changes our interpretation of the results. While the cues appeared simultaneously, subjects could immediately extract information about the target location from the visual cue, whereas the haptic cue was only felt during movement with a magnitude dependent on the perpendicular distance within the force channel. Prior work provides evidence that humans are capable of integrating noisy sensory information over time when estimating hand position (Saunders and Knill 2004; Wolpert et al. 1995) and making decisions (Gold and Shadlen 2007). In our study, subjects were able to appropriately use the haptic information, although the temporal delay could have prevented them from extracting sufficient information from the haptic cue. This would have likely resulted in increased weighting of the visual cue, which was inconsistent with our data. On the other hand, a recent study suggests that differences in the time course of the acquisition of sensory information can affect whether cues are even integrated (Plaisier et al. 2014). In forming a percept of surface orientation, visual and haptic cues were only combined when the exploration mode (parallel vs. serial) was the same. In our study, the additional cognitive component about cue correspondence may explain why both the visual and haptic cues were used to locate the hidden target (Sutter et al. 2014).

Considering the physical dynamics of the haptic cue, its effect on movement could have presented itself as a force perturbation, given a large discrepancy between the direction of movement and the force channel. During the relatively fast reaches, subjects may not have had enough time to appropriately correct for such perturbations. To quantify the effect of the force channel on a subject’s final position, two of the original subjects performed a supplemental experiment (see Appendix). Results show a small bias of approximately 0.1 cm towards the haptic cue. In the original experiment, the distance between a subject’s final position and the haptic centroid covered a much larger spread (considering all analysed trials across all subjects: 10th percentile = 0.04 cm, 90th percentile = 1.0 cm). Thus, any bias on the endpoint position due to the haptic cue cannot explain the observed reweighting.

### Implications for haptic assistance

The ability of the brain to integrate both naturally occurring and augmented cues, even if erroneous, is promising for the development of intelligent haptic assistance systems. Recent research has explored different forms of haptic assistance (e.g. virtual fixtures, haptic guidance, haptic shared control) to physically assist a human in performing a task, ranging from cooperative manipulation to teleoperation to vehicle control (Rosenberg 1993; Abbott et al. 2007; Marchal-Crespo and Reinkensmeyer 2008; Abbink et al. 2012; Passenberg et al. 2013). Assistance via the haptic modality is intuitive and enables the human to quickly react to or overrule the suggested commands. Previous studies typically assume perfect knowledge of the task and environment, upon which the haptic assistance is based. Realistically, haptic assistance systems need to operate in unstructured and dynamic environments, where sensor noise and model inaccuracies can result in erroneous assistance (van Oosterhout et al. 2015). The implications of these results are favourable for haptic assistance under such practical conditions; apparently, haptic assistance does not need to be perfectly accurate to be useful, given that humans can appropriately adjust their trust in the haptic cue depending on the statistics of its error history.

## Appendix

The physical dynamics of the haptic cue used in our study act not only as a sensory cue, but can also influence a subject’s movement. To disentangle these two effects, a supplemental experiment was performed to quantify the latter—the effect of the haptic cue on a subject’s final position.

Two of the original subjects participated in the supplemental experiment, which used the same target-hitting task. First, subjects performed 50 trials with the true location of the target shown visually (similar to the familiarization block). Next, they performed 50 trials with the true location of the target shown, in addition to the haptic cue with trial-by-trial random errors (similar to the haptic familiarization block, *σ*_Hc,t_ = 1 cm). For all trials, subjects were instructed to hit the visually displayed target, thus overruling the haptic cue. The target error (distance between the subject’s final position and the target position) was measured for each trial.

With the addition of the force channel, there was a slight bias of approximately 0.1 cm towards the haptic centroid (Table 2). In the original experiment, the distance between a subject’s final position and the haptic centroid covered a much larger spread than this bias (considering all analysed trials across all subjects: 10th percentile = 0.04 cm, 90th percentile = 1.0 cm). Thus, any bias on the final position due to the physical dynamics of the haptic cue cannot explain the reweighting observed in the main experiment.

**Table 2:** Supplemental experiment results

Subject	True target		True target + H cue		
	μ (cm)	σ (cm)	μ (cm)	σ (cm)	μ bias towards H (cm)
1	−0.027	0.42	−0.035	0.31	0.10
2	−0.039	0.51	0.012	0.28	0.12

Measurements for two subjects when the true target was visible, without the haptic cue (columns 2, 3) and with the haptic cue (columns 4, 5, 6). Haptic cue contained trial-by-trial random errors (*σ*_Hc,t_ = 1 cm). Target error: subject’s final position with respect to the true target, where the direction of the haptic cue could be positive or negative. Bias towards H: subject’s final position with respect to the true target, with the direction of the haptic cue taken as positive

The standard deviation of the target error also decreased slightly with the addition of the haptic cue (Table 2). This increased precision is in agreement with some of the subjects’ comments that aiming became easier with the (albeit erroneous) haptic cue, because they could “lean” against the force channel for support. The increased movement precision with the haptic cue may have affected the measured target error variability in the combined cue conditions, although its effect is not entirely clear.
